# Respiratory chain gene mutations associated with global phylogenetic clustering of drug-resistant *Mycobacterium tuberculosis* revealed by whole-genome sequencing

**DOI:** 10.3389/fimmu.2026.1724194

**Published:** 2026-05-20

**Authors:** Qiang Ji, Yawei Hou, Yameng Li

**Affiliations:** 1 Shandong University of Traditional Chinese Medicine, Jinan, Shandong, China; 2 Department of Traditional Chinese Medicine, Qilu Second Hospital of Shandong University, Jinan, Shandong, China; 3 Department of Respiratory and Critical Care Medicine, The Third Affiliated Hospital of Shandong First Medical University (Affiliated Hospital of Shandong Academy of Medical Sciences), Jinan, Shandong, China

**Keywords:** multidrug-resistant, *Mycobacterium tuberculosis*, respiratory chain, transmission, whole-genome sequencing

## Abstract

**Background:**

The regulatory mechanism of respiratory chain in *Mycobacterium tuberculosis* (*M. tuberculosis*) remains to be elucidated, and limited evidence about the effect of gene mutations in the respiratory chain on multidrug-resistant (MDR) isolates was reported.

**Objective:**

To elucidated the effect of gene mutations in the respiratory chain on MDR isolates.

**Methods:**

Whole-genome sequencing was performed on analyzed strains of *M. tuberculosis*. Random forest, gradient boosting decision tree and generalized linear mixed models were employed to identify mutations in respiratory chain genes that contribute to the phylogenetic clustering and development of MDR isolates.

**Results:**

Overall, a total of 13402 isolates of *M. tuberculosis* were included in the study. 4051 (30.09%) isolates showed MDR, and 1044 (7.76%) isolates were classified as single‐drug resistance (SDR). The results showed that the single nucleotide polymorphisms (SNPs) of *atpH* A428G, *cydA* C942A, *qcrA* G181C, *nuoF* G66C, *qcrB* G1250T, *nuoA* G82C, and *nuoG* A1422G A1810G were significantly associated with phylogenetic clustering of MDR isolates. The SNPs of *ndhA* G1000A, *atpH* C73G A428G, *cydA* C942A, *qcrA* G181C, *qcrB* G55A, *nuoG* A1422G, *nuoJ* G115A, *nuoN* G1084T, *cydB* T126C, *nuoA* G82C, *nuoB* G490C, *nuoF* C171T, and *nuoK* C73T were significantly associated with formation of MDR isolates.

**Conclusion:**

Our study proposed that SNPs in genes of the respiratory chain were related to the phylogenetic clustering and development of MDR isolates, which provides new insights for preventing the phylogenetic clustering and development of MDR isolates. These insights provide valuable information for the development of potential therapeutic targets for MDR isolates.

## 1 Introduction

Tuberculosis (TB), caused by *Mycobacterium tuberculosis* (*M. tuberculosis*), is posing a great challenge to global public health, especially with the emergence of multidrug-resistant TB (MDR-TB). According to the World Health Organization (WHO), an estimated 450,000 new rifampicin-resistant TB (RR-TB) occurred globally in 2021, of which 78% were MDR-TB (1).

The respiratory chain genes of *M. tuberculosis* include several genes encoding proteins. These proteins enable *M. tuberculosis* to adapt to different environmental conditions and maintain its growth and reproduction by maintaining energy supply, redox balance and cellular respiratory function. Among them, the main respiratory chain genes include NADH dehydrogenase, cytochrome oxidase and ATP synthase (2).

The expression of respiratory chain genes in *M. tuberculosis* is influenced by many environmental cues, one of which is oxygen level. In a hypoxic environment, *M. tuberculosis* can adapt to hypoxic conditions by regulating the expression of respiratory chain genes, thus maintaining its own survival (3). Changes in these respiratory chain genes may lead to changes in the metabolic pathway of drugs in bacteria, thus reducing the effectiveness of drugs. In recent years, the Food and Drug Administration (FDA) has approved two electron transfer chain inhibitors-an ATPase inhibitor bedaquiline and a nitric oxide donor and respiratory poison pretomanid-for the treatment of multidrug-resistant *M. tuberculosis* (4). However, limited studies have been conducted on the role of respiratory chain gene mutations in MDR-TB transmission. Therefore, it becomes crucial to gain a deeper understanding of the transmission mechanisms of MDR-TB and their associations with mutations in respiratory chain genes.

With the development of sequencing technology, whole-genome sequencing (WGS) is increasingly becoming the preferred method for studying drug-resistant pathogens in health care systems. In our study, WGS was used to investigate the impact of gene mutations in the respiratory chain on the global transmission of MDR isolates. Specifically, the genome cluster was used to represent the transmission of *M. tuberculosis*.

## 2 Materials and methods

### 2.1 Sample collection

Between 2011 and 2018, we collected 1,550 cases of individuals diagnosed with *M. tuberculosis* infection from two major medical institutions in China: the Shandong Public Health Clinical Research Center (SPHCC) and the Weifang Respiratory Disease Hospital (WRDH). Inclusion criteria consisted of individuals confirmed based on clinical symptoms, positive *M. tuberculosis* culture results, or molecular biology tests (such as PCR). This study specifically excluded patients who had previously undergone evaluation and were currently receiving treatment for culture-positive *M. tuberculosis*, as well as those with severe comorbidities or lacking follow-up data, to ensure the representativeness and validity of the sample. All data were obtained from electronic health records and verified by experienced medical professionals to ensure accuracy. Additionally, these two institutions are located in high-incidence areas, which allows for a better reflection of the local epidemiological characteristics of *M. tuberculosis* infection, thereby enhancing the diversity and breadth of the sample.

### 2.2 DNA extraction and sequencing

*M. tuberculosis* isolates were cultured on Löwenstein-Jensen (L-J) medium at 37°C for four weeks, followed by genomic DNA extraction using the cetyltrimethylammonium bromide (CTAB) method (Sigma-Aldrich). DNA quality was confirmed by Nanodrop (A260/A280 ratio: 1.8-2.0) and agarose gel electrophoresis. After excluding 105 isolates (103 due to failed extraction and 2 lost during processing), 1,445 isolates were sequenced on an Illumina HiSeq 4000 system (5). This dataset was merged with 11,957 publicly available global isolates (3, 6–13) All reads were mapped to the H37Rv genome using BWA-MEM (v0.7.17). To qualify for analysis, samples were required to have≥98% coverage and a minimum depth of 20x (14), a final set of 13,402 genomes was analyzed. Data are available under NCBI BioProject PRJNA1002108, with sample details in **Supplementary Tables 1**, **2**.

### 2.3 Single nucleotide polymorphism analysis

The variant calling workflow began by aligning sequences to the H37Rv reference genome. Initial variant calling was performed per sample using FreeBayes (v1.3.2) with the filter “FMT/GT = ‘1/1’ && QUAL >= 100 && FMT/DP >= 10 && (FMT/AO)/(FMT/DP) >= 0.9”. The resulting individual VCF files were then pooled and merged using Bcftools (v1.15.1). To further eliminate false positives caused by misaligned reads, we employed Samclip (version 0.4.0) to identify and remove variants supported primarily by soft-clipped alignments. Prior to variant calling, SAMtools (version 1.15) was used for essential processing of the BAM files, including sorting, indexing, and duplicate marking. Subsequently, a biological filter was applied. Single Nucleotide Polymorphisms (SNPs) situated within repetitive genomic regions, encompassing polymorphic GC-rich sequences associated with PE/PPE genes and tandem repeats identified using Tandem Repeat Finder (version 4.09) and RepeatMasker (version 4.1.2-P1) (15, 16), were systematically excluded from our analysis. The final high-confidence SNP set was functionally annotated using SnpEff (version 4.1l), and all subsequent data handling was performed using custom scripts in the Python programming language (4).

### 2.4 Drug resistance prediction

To identify drug resistance mutations, we compared known indels and SNPs using TBProfiler (version 2.8.12) and the tuberculosis database (TBDB) (17, 18). We then searched for genotypic markers of drug resistance mutations in both first-line drugs (such as isoniazid, rifampicin, pyrazinamide, ethambutol, and streptomycin) and second-line drugs (such as ethionamide, quinolones, amikacin, capreomycin, and kanamycin), using a set of genetic polymorphisms. Mutations that were not correlated with phenotypic drug resistance were excluded as markers of genetic drug resistance (2). For more information about the mutations detected as molecular resistance predictions in 13402 isolates, please refer to **Supplementary Table 3**.

### 2.5 Phylogenetic analysis

The isolates were classified into different lineages according to Coll et al. (19) (**Supplementary Tables 1**, **2**). The phylogenetic relationships among isolates were inferred using a maximum-likelihood approach. A core genome alignment of high-quality SNPs relative to the *M. tuberculosis* H37Rv reference genome (NC_000962.3) was used as the input for IQ-TREE. This alignment was generated by first mapping the sequencing reads of all 13,402 isolates to the reference genome using BWA-MEM, followed by variant calling. The resulting variant calls were filtered to exclude repetitive regions and phages. Subsequently, a site-aligned SNP alignment was created, which constituted the core genome alignment used for phylogenetic reconstruction. The maximum-likelihood tree was constructed with IQ-TREE (version 1.6.12) using the Jukes-Cantor (JC) nucleotide substitution model and a gamma model of rate heterogeneity, with branch support assessed from 1000 bootstrap replicates. *Mycobacterium canettii CIPT 140010059* was used as the outgroup. The final tree was visualized and annotated using iTOL (https://itol.embl.de/).

### 2.6 Propagation analysis

Cluster analysis was utilized to investigate the influence of respiratory chain gene mutations on the transmission of *M. tuberculosis*. Clustering was defined as a grouping of isolates exhibiting less than 12 SNPs among each other (20). **Supplementary Tables 1**, **2**.

### 2.7 Acquisition of respiratory chain genes

A total of 29 respiratory chain genes were obtained according to NCBI. Python was utilized to detect mutations in genes associated with respiratory chain genes, **Supplementary Table 4**.

### 2.8 Statistical analysis

Descriptive statistics were presented as means and standard errors for numerical variables, and frequencies and percentages for categorical variables. To enhance statistical robustness and mitigate overfitting from rare variants, all mutations observed in fewer than ten instances across the entire dataset were excluded from subsequent association analyses. To evaluate the impact of respiratory chain gene mutations on the formation of MDR-TB phylogenetic clustering, we employed a representative-strain sampling strategy to address statistical non-independence. Specifically, a single strain was randomly selected from each identified MDR phylogenetic clustering to serve as its representative. These representative strains were then used for subsequent statistical analysis. All statistical analyses were performed using R (version 4.2.3; R Foundation for Statistical Computing). Generalized linear mixed models (GLMMs) were implemented using the lme4 package (version 1.1.35.1). Specifically, lineage and sampling location were included as random intercepts in the model to control for their confounding effects. The p-values obtained from the GLMM analyses for all SNPs were adjusted for multiple testing using the Benjamini-Hochberg false discovery rate (FDR) correction. An FDR-adjusted *p*-value (q-value) of < 0.05 was considered statistically significant. The random forest and gradient boosting decision tree were implemented using Python 3.7.4 with scikit-learn (version 1.2.2). The dataset was randomly assigned to either the training set or test set in a 7:3 ratio. Model performance was evaluated on the held-out test set based on accuracy and area under the receiver operating characteristic curve (AUC). In the Random Forest classification, feature importance was measured using the Gini impurity-based importance score, which ranges from 0 to 1 (21). For the Gradient Boosting model, the Gain importance metric was used (22). In addition, our analysis to control for the confounding influence of both bacterial lineage and geographical origin.

## 3 Results

### 3.1 Sample description

**Figure 1:**
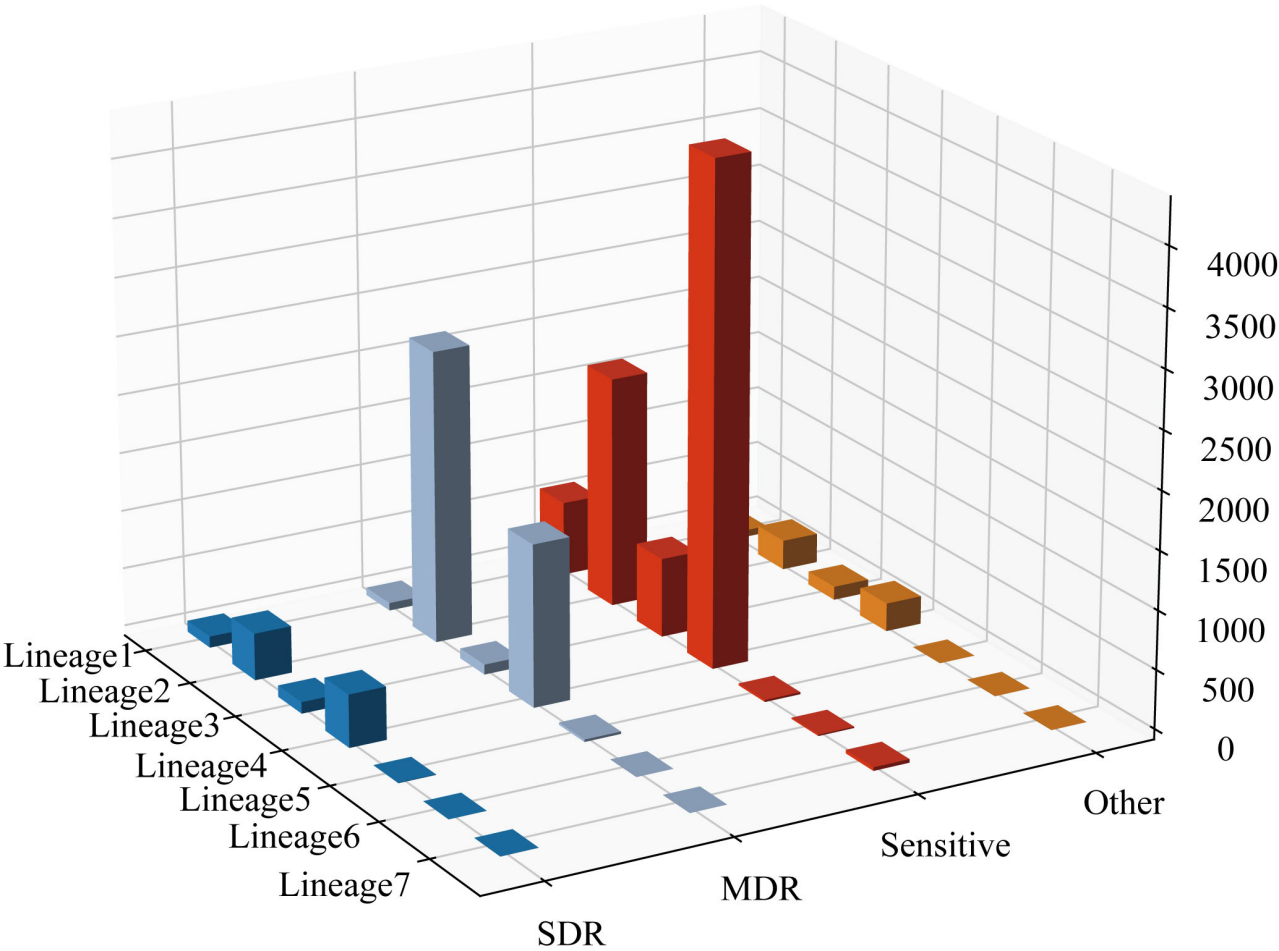
Distribution of drug resistance in different lineages.

We included a total of 13,402 isolates of *M. tuberculosis* worldwide in the study. Most isolates belonged to lineage4 (n=6382,47.41%), followed by lineage2 (n=5123,38.06%). We found that these isolates primarily come from Eastern Asia with a total of 3,160 (23.48%), followed by Eastern Africa with 1,657 (12.31%), and Northern America with 1,637 (12.16%). Among these isolates, 4,051 (30.09%) showed MDR, while 1,044 isolates (7.76%) were classified as single drug resistance (SDR). For further details regarding the *M. tuberculosis* isolates see text in **Figure 1** and **Table 1**.

**Table 1:** The characteristics of *Mycobacterium tuberculosis* isolates worldwide.

Characteristic	Number (%)
Lineage	
Lineage1	851(6.35)
Lineage2	5123(38.23)
Lineage3	969(7.23)
Lineage4	6382(47.62)
Lineage5	38(0.28)
Lineage6	10(0.07)
Lineage7	29(0.22)
Drug characteristics	
SDR	1044(7.76)
MDR	4051(30.09)
Sensitive	7645(56.79)
Other	662(4.92)
MDR characteristics	
Clustered	1987(49.05)
No-clustered	2064(50.95)

MDR, multidrug-resistant (resistance to at least isoniazid and rifampicin); SDR, single drug-resistant (resistance to exactly one first-line drug); Other, isolates exhibiting other resistance patterns, including monoresistance to drugs other than isoniazid or rifampicin, or polyresistance not meeting the criteria for MDR.

### 3.2 MDR isolates transmission and respiratory chain gene mutation

**Figure 2:**
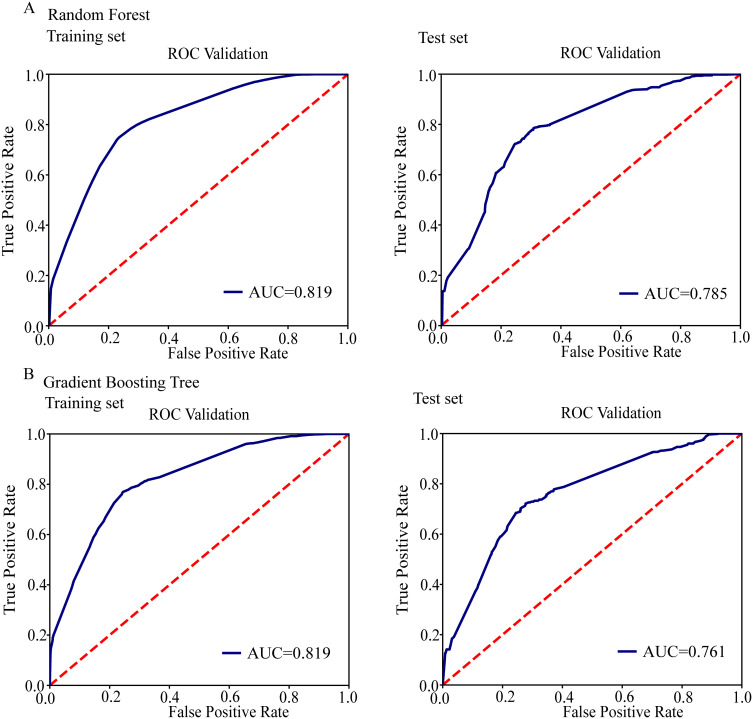
ROC curve analysis was conducted to evaluate the performance of models used for multidrug resistance cluster analysis. **(A)** ROC analysis showing the performance of the random forest model. **(B)** ROC analysis showing the performance of the gradient boosting decision tree.

Compared with non-clustered isolates of MDR, we analyzed the relationship between 62 SNPs and clustered isolates of MDR. The GLMM showed that 10 SNPs were found to be statistically significant for clustering (*p* < 0.05), among which six nonsynonymous SNPs and two synonymous SNPs were positively correlated with clustering, including *atpH* (*Rv1307*, A428G, Gln143Arg; OR, 106.485, 95%CI, 41.449-273.566), *cydA* (*Rv1623c*, C942A, Ile314Ile; OR, 6.11, 95%CI, 2.386-15.646), *qcrA* (*Rv2195*,G181C,Glu61Gln, OR,21.585, 95%CI, 5.227-89.144), *nuoF* (*Rv3150*, G66C,Trp22Cys, OR,4.938, 95%CI, 1.011-24.12), *qcrB* (*Rv2196*, G1250T,Arg417Leu, OR, 7.272, 95%CI, 3.221-16.418), *nuoA* (*Rv3145*, G82C,Val28Leu, OR,1.906, 95%CI, 1.035-3.51), and *nuoG* (*Rv3151*,A1422G,Ile474Met, OR, 1.801, 95%CI, 1.084-2.994; A1810G, Thr60, OR, 4.229, 95%CI, 1.947-9.185), see **Table 2**. Two prediction models were established using random forest and gradient boosting decision tree, **Figure 2**. We found that *atpH* A428G, *cydA* C942A, *qcrB* G1250T, *nuoA* G82C, *qcrA* G181C, *nuoF* G66C, and *nuoG* A1422G were among those which contributed most to the random forest and gradient boosting decision tree. The results indicated that SNPs of *atpH* A428G, *cydA* C942A, *qcrB* G1250T, *nuoA* G82C, *qcrA* G181C, *nuoF* G66C, and *nuoG* A1422G A1810G increased the risk of phylogenetic clustering among MDR isolates. See **Supplementary Tables 5**, **9** for details.

**Table 2:** Generalized linear mixed model analysis on clustered and non-clustered isolates of MDR isolates.

Gene	Position	SNP	Amino acid changes	*p-*Value	OR (95%CI)
hycE	95697	C284T	Pro95Leu	0.995	NA
ndhA	471612	A1028G	Tyr343Cys	0.417	1.695(0.474-6.056)
ndhA	471640	G1000A	Ala334Thr	0.178	0.204(0.02-2.068)
ndhA	471666	T974C	Met325Thr	0.996	NA
ndhA	472236	C404A	Ala135Glu	0.864	0.915(0.328-2.55)
atpB	1460552	G309T	Trp103Cys	0.805	0.843(0.217-3.269)
atpB	1460907	T664C	Phe222Leu	0.998	NA
atpH	1461915	C73G	Leu25Val	0.222	2.457(0.58-10.403)
atpH	1462152	C310A	Arg104Ser	0.710	1.121(0.613-2.052)
atpH	1462270	A428G	Gln143Arg	<0.001	106.485(41.449-273.566)
atpH	1462787	C945A	Asp315Glu	0.268	1.651(0.68-4.007)
atpA	1463498	G271C	Val91Leu	0.395	1.895(0.435-8.257)
atpA	1464859	G1632A	Ala544Ala	0.272	0.702(0.373-1.32)
cydA	1824946	C942A	Ile314Ile	<0.001	6.11(2.386-15.646)
qcrC	2458368	G816C	Leu272Leu	0.521	0.633(0.157-2.56)
qcrA	2458572	G181C	Glu61Gln	<0.001	21.585(5.227-89.144)
qcrA	2458639	T248C	Val83Ala	0.894	0.925(0.292-2.931)
qcrA	2459162	G771A	Glu257Glu	0.797	0.835(0.211-3.3)
qcrB	2459732	G55A	Glu19Lys	0.998	NA
qcrB	2460254	T577G	Trp193Gly	0.383	0.604(0.194-1.878)
qcrB	2460927	G1250T	Arg417Leu	<0.001	7.272(3.221-16.418)
ctaD	3403250	G1672A	Ala558Thr	0.991	NA
ctaD	3404376	G546C	Thr182Thr	0.030	0.626(0.41-0.955)
nuoA	3511763	G82C	Val28Leu	0.038	1.906(1.035-3.51)
nuoD	3514512	G1175C	Gly392Ala	0.708	1.108(0.649-1.893)
nuoE	3515307	G651A	Gln217Gln	0.419	0.683(0.271-1.721)
nuoF	3515477	G66C	Trp22Cys	0.048	4.938(1.011-24.12)
nuoF	3515582	C171T	Ser57Ser	0.843	1.07(0.548-2.092)
nuoF	3516342	C931T	Leu311Leu	0.511	0.608(0.138-2.683)
nuoG	3517413	C668T	Ala223Val	0.990	NA
nuoG	3518167	A1422G	Ile474Met	0.023	1.801(1.084-2.994)
nuoG	3518441	T1696C	Leu566Leu	0.191	1.849(0.735-4.65)
nuoG	3518555	A1810G	Thr604Ala	<0.001	4.229(1.947-9.185)
nuoH	3519732	C451T	Leu151Leu	0.096	0.143(0.014-1.41)
nuoH	3519774	G493A	Val165Ile	0.215	0.605(0.274-1.338)
nuoI	3520977	G471C	Leu157Leu	0.369	0.741(0.386-1.424)
nuoI	3521044	A538G	Thr180Ala	0.998	NA
nuoJ	3521253	G115A	Val39Ile	0.979	1.007(0.615-1.648)
nuoL	3523741	C1508A	Thr503Asn	0.540	1.55(0.381-6.304)
nuoM	3524528	G397T	Gly133Cys	0.302	0.573(0.199-1.649)
nuoM	3524903	G772A	Ala258Thr	0.024	0.263(0.083-0.839)
nuoM	3525374	A1243G	Thr415Ala	0.991	NA
nuoN	3526873	G1084T	Ala362Ser	0.518	1.14(0.767-1.694)
nuoN	3526987	G1198A	Gly400Ser	0.147	4.745(0.578-38.941)
hycE	95591	G178T	Glu60*	0.449	0.58(0.142-2.376)

SNP, single nucleotide polymorphism; OR, odd ratio; CI, confident interval; NA, not available.

### 3.3 SDR isolates, MDR isolates and respiratory chain gene mutation

Compared with SDR isolates, we analyzed the relationship between 77 SNPs and development of MDR isolates. The GLMM showed that 16 SNPs were statistically significant for the development of MDR isolates (*p* < 0.05), among which seven nonsynonymous SNPs and two synonymous SNPs were positively correlated with the development of MDR isolates, including *ndhA* (*Rv0392c*, G1000A,Ala334Thr), *atpH* (*Rv1307*, C73G,Leu25Val; A428G,Gln143Arg), *cydA* (*Rv1623c*, C942A,Ile314Ile), *qcrA* (G181C,Glu61Gln), *qcrB* (*Rv2196*, G55A,Glu19Lys), *nuoG* (*Rv3151*,A1422G,Ile474Met), *nuoJ* (*Rv3154*, G115A,Val39Ile), and *nuoN* (*Rv3158*, G1084T,Ala362Ser), see **Table 3**. Two prediction models were established using random forest and gradient boosting decision tree, **Supplementary Figure 1**. We found that *ndhA* G1000A, *atpH* C73G A428G, *cydA* C942A, *qcrA* G181C, *qcrB* G55A, *nuoG* A1422G, *nuoJ* G115A, and *nuoN* G1084T also contributed most to the random forest and gradient boosting decision tree. The results indicated that *ndhA* G1000A, *atpH* C73G A428G, *cydA* C942A, *qcrA* G181C, *qcrB* G55A, *nuoG* A1422G, *nuoJ* G115A, and *nuoN* G1084T were associated with MDR isolates compared with SDR isolates, see **Supplementary Tables 6**, **10** for details.

**Table 3:** Analysis of generalized linear mixed model of MDR and SDR isolates.

Gene	Position	SNP	Amino acid changes	*p-*Value	OR(95%CI)
hycE	95591	G178T	Glu60*	0.011	0.279(0.105-0.742)
hycE	95697	C284T	Pro95Leu	0.999	NA
ndhA	471469	G1171A	Gly391Arg	0.998	NA
ndhA	471612	A1028G	Tyr343Cys	0.319	0.516(0.141-1.884)
ndhA	471640	G1000A	Ala334Thr	0.001	23.22(3.722-144.873)
ndhA	471646	G994C	Gly332Arg	0.961	1.046(0.172-6.35)
ndhA	471666	T974C	Met325Thr	0.996	NA
ndhA	472236	C404A	Ala135Glu	0.600	1.763(0.214-14.515)
ndhA	472471	C169T	Gln57*	0.998	NA
atpB	1460552	G309T	Trp103Cys	1.000	NA
atpB	1460907	T664C	Phe222Leu	0.772	0.781(0.147-4.142)
atpH	1461915	C73G	Leu25Val	<0.001	16.216(4.782-54.987)
atpH	1462152	C310A	Arg104Ser	0.869	1.067(0.495-2.302)
atpH	1462270	A428G	Gln143Arg	<0.001	90.468(11.882-688.799)
atpH	1462787	C945A	Asp315Glu	0.427	1.925(0.386-9.612)
atpH	1462845	G1003A	Asp335Asn	0.076	3.955(0.875-17.884)
atpA	1463498	G271C	Val91Leu	0.999	NA
atpA	1464859	G1632A	Ala544Ala	0.932	0.953(0.319-2.849)
cydB	1823378	G1023A	Leu341Leu	<0.001	0.104(0.033-0.329)
cydB	1823579	G822A	Leu274Leu	0.550	0.456(0.035-5.907)
cydB	1823738	G663A	Leu221Leu	0.120	3.337(0.737-15.106)
cydB	1823798	C603T	Ala201Ala	1.000	NA
cydA	1824800	G1088A	Arg363His	0.997	NA
cydA	1824946	C942A	Ile314Ile	<0.001	5.954(2.458-14.419)
qcrC	2458368	G816C	Leu272Leu	0.999	NA
qcrA	2458572	G181C	Glu61Gln	<0.001	22.783(4.743-109.438)
qcrA	2458639	T248C	Val83Ala	0.280	3.232(0.39-26.809)
qcrA	2459162	G771A	Glu257Glu	0.997	NA
qcrB	2459732	G55A	Glu19Lys	0.038	4.491(1.094-18.427)
qcrB	2460254	T577G	Trp193Gly	0.087	0.314(0.084-1.177)
qcrB	2460927	G1250T	Arg417Leu	0.879	1.094(0.346-3.455)
ctaD	3403250	G1672A	Ala558Thr	0.340	0.394(0.058-2.65)
ctaD	3404376	G546C	Thr182Thr	0.001	0.521(0.351-0.773)
nuoA	3511763	G82C	Val28Leu	0.452	0.754(0.362-1.569)
nuoB	3512566	G490C	Ala164Pro	0.749	1.174(0.442-3.118)
nuoD	3514512	G1175C	Gly392Ala	0.018	0.529(0.313-0.896)
nuoE	3515307	G651A	Gln217Gln	0.469	1.544(0.479-4.975)
nuoF	3515467	C56T	Pro19Leu	0.996	NA
nuoF	3515477	G66C	Trp22Cys	0.999	NA
nuoF	3515582	C171T	Ser57Ser	0.071	2.863(0.92-8.915)
nuoF	3516299	C888A	Gly296Gly	0.908	0.882(0.106-7.32)
nuoF	3516342	C931T	Leu311Leu	0.576	1.422(0.416-4.862)
nuoG	3517413	C668T	Ala223Val	0.071	0.143(0.017-1.167)
nuoG	3518089	T1344C	Gly448Gly	0.998	NA
nuoG	3518167	A1422G	Ile474Met	<0.001	2.477(1.554-3.949)
nuoG	3518441	T1696C	Leu566Leu	0.607	1.47(0.341-6.33)
nuoG	3518555	A1810G	Thr604Ala	0.004	0.147(0.04-0.539)
nuoH	3519732	C451T	Leu151Leu	1.000	NA
nuoH	3519774	G493A	Val165Ile	0.216	2.475(0.593-10.33)
nuoH	3520475	G1194A	Pro398Pro	1.000	NA
nuoI	3520977	G471C	Leu157Leu	0.872	0.95(0.513-1.761)
nuoI	3521044	A538G	Thr180Ala	0.139	10.848(0.47-250.51)
nuoJ	3521253	G115A	Val39Ile	0.001	6.994(2.121-23.062)
nuoL	3523741	C1508A	Thr503Asn	0.999	NA
nuoM	3524528	G397T	Gly133Cys	<0.001	0.205(0.105-0.398)
nuoM	3524581	C450T	Ile150Ile	0.770	0.659(0.041-10.623)
nuoM	3524903	G772A	Ala258Thr	0.004	0.242(0.092-0.636)
nuoM	3525374	A1243G	Thr415Ala	0.759	1.372(0.184-10.246)
nuoN	3526021	G232T	Val78Leu	0.088	0.184(0.027-1.271)
nuoN	3526137	T348G	Ala116Ala	0.368	0.586(0.185-1.861)
nuoN	3526873	G1084T	Ala362Ser	0.049	1.96(1.006-3.819)
nuoN	3526884	G1095A	Pro365Pro	0.442	2.743(0.212-35.424)
nuoN	3526987	G1198A	Gly400Ser	1.000	NA

SNP, single nucleotide polymorphism; OR, odd ratio; CI, confident interval; NA, not available.

### 3.4 Sensitive isolates, MDR isolates and respiratory chain gene mutation

Compared with sensitive isolates, we analyzed the relationship between 153 SNPs and development of MDR isolates. The GLMM showed that 23 SNPs were statistically significant for the development of MDR isolates (*p* < 0.05), among which eight nonsynonymous SNPs and three synonymous SNPs were positively correlated with the development of MDR isolates, including *ndhA* (*Rv0392c*, G1000A, Ala334Thr), *atpA* (*Rv1308*, G271C, Val91Leu), *cydA* (*Rv1623c*, C942A, Ile314Ile; G1088A, Arg363His), *cydB* (*Rv1622c*, T126C, Asp42Asp), *nuoA* (*Rv3145*, G82C, Val28Leu), *nuoB* (*Rv3146*, G490C, Ala164Pro), *nuoF*(C171T,Ser57Ser), *nuoG* (*Rv3151*, A1422G, Ile474Met), *nuoK* (*Rv3155*, C73T, Arg25Cys), and *nuoN* (*Rv3158*, G1084T, Ala362Ser), see **Supplementary Table 8**. Two prediction models were established using random forest and gradient boosting decision tree, **Supplementary Figure 2**. We found that *ndhA* G1000A, *cydA* C942A G1088A, *cydB* T126C, *nuoA* G82C, nuoB G490C, *nuoF* C171T, *nuoG* A1422G, *nuoK* C73T and *nuoN* G1084T also contributed most to the random forest and gradient boosting decision tree. However, there was no contribution of *atpA* G271C to the gradient boosting decision tree model. The results indicated that compared with sensitive isolates, *ndh*A G1000A, *cydA* C942A G1088A, *cydB* T126C, *nuoA* G82C, *nuoB* G490C, *nuoF* C171T, *nuoG* A1422G, *nuoK* C73T and *nuoN* G1084T increased the risk of development of MDR isolates, see **Supplementary Tables 7**, **11** for details.

## 4 Discussion

Respiratory chain plays a crucial role in the growth of MDR isolates. To investigate the relationship between MDR development, MDR phylogenetic clustering and respiratory chain gene mutation, we included 13402 isolates of *M. tuberculosis* worldwide and 29 respiratory chain genes. Most of the MDR isolates belonged to lineage2 (n=2498,61.7%; Beijing lineage), followed by lineage 4 (n=1386,34.2%; European lineage). Most of the clustered isolates of MDR also belonged to lineage2 (n=1617,59.6%), which indicated that the main isolates of MDR phylogenetic clustering belonged to lineage2 worldwide. In addition, the phylogenetic tree of *M. tuberculosis* isolates from China was established, **Supplementary Figure 3**.

Although the selected SNPs did not exhibit the absolute highest scores, they were prioritized based on a combination of their high feature importance scores and their well-established biological relevance to drug resistance and respiratory function in known genes. This multi-criteria approach ensures a focus on statistically significant mutations with clear biological implications. Our results revealed that the synonymous SNP of *cydA* C942A and the non-synonymous SNP of *nuoG* A1422G were not only related to the risk of MDR phylogenetic clustering, but also related to the development of MDR.

Our results revealed that the synonymous SNP *cydA* C942A and the non-synonymous SNP *nuoG* A1422G were significantly associated with both MDR transmission clusters and MDR strains. *CydA* encodes the cytochrome bd ubiquinol oxidase subunit I, an enzyme less prone to inhibition by oxidative stress, enabling aerobic metabolism to continue under adverse conditions. Early studies have shown that *cydA* plays a key role in the growth and metabolism of mycobacterium tuberculosis. By deleting or mutating the *cydA* gene, the assembly and activity of cytochrome bd enzyme can be affected, resulting in a decrease in the bacteria’s ability to utilize oxygen (23–25). In addition, While *cydA* mutations have been linked to altered susceptibility to certain experimental compounds (26), their role in resistance to conventional anti-tuberculosis drugs is less clear. In our study, we found that the *cydA* C942A mutation was significantly associated with the transmission of MDR strains, suggesting a potential fitness advantage that is independent of conventional drug resistance. Notably, we detected non-synonymous SNPs in *qcrA* and *qcrB*, which encode subunits of the cytochrome bc1 complex (Complex III). While these variants did not rank as the top features in our model, their presence is biologically intriguing. The cytochrome bc1 complex is a key component of the proton-motive respiratory chain and a target of the candidate drug Q203. Mutations in *qcrA/B* have been previously linked to resistance to this class of inhibitors. Their detection here, albeit at a lower frequency, hints at a potential diversification of respiratory adaptations in circulating MDR strains (27, 28). Our results also confirmed that both synonymous and non-synonymous mutations can affect the transmission of *M. tuberculosis*, indicating that synonymous mutations in respiratory chain of *M. tuberculosis* are not all neutral mutations. *M. tuberculosis nuoG* encodes a subunit of the NAD(P)H dehydrogenases comprise type 1 (NDH-1), which is necessary for suppression of reactive oxygen species formed by the host macrophage NOX2 complex and thus inhibits TNF-mediated apoptosis induction (29). We found that the SNP *nuoG* A1422G may impair the activity, expression, or assembly of NDH-1, leading to disorders of the respiratory chain and energy metabolism in *M. tuberculosis*. The potential physiological consequences of this metabolic defect, such as its impact on bacterial fitness and drug susceptibility, warrant further investigation (30). In addition, we found that the SNP of nuoG A1810G was also associated with the risk of *M. tuberculosis* transmission, which may change the function or stability of NDH-1 and affect the bacterial respiratory chain and energy metabolism. However, to deeply understand the mechanism of action of *nuoG* SNP in the development and transmission of multidrug-resistant tuberculosis, more research is needed to further verify this association.

Moreover, our investigation revealed a noteworthy finding: the non-synonymous SNP A428G in the *atpH* gene exhibited a significant association with MDR strains. It is crucial to note that the *atpH* gene encodes a vital subunit of ATP synthase F0, an integral component intricately involved in the intricate process of energy production within bacterial cells (31). Regrettably, the research conducted on *atpH* has been rather limited, leaving us with mere speculations that SNP variations could potentially disrupt the structural integrity, functional efficacy, or even the stability of ATP synthase F0, thereby leading to profound metabolic disturbances. Furthermore, our analysis identified additional non-synonymous SNPs, specifically G1000A in *ndhA* and G1084T in *nuoN*, which exhibited a notable correlation with the risk of MDR development. These two genes, *ndhA* and *nuoN*, encode pivotal subunits of NADH dehydrogenase, which collectively orchestrate the intricate electron transfer process of NADH. This essential process plays a critical role in supporting both bacterial energy generation and cellular metabolism (32).

Despite these intriguing findings, our understanding of respiratory chain genes in *M. tuberculosis* remains preliminary. The functional implications of the identified SNPs on protein stability and activity, as well as their precise role in MDR transmission and development, require further validation. A critical next step will be to compare our strains against global databases to assess the generalizability of our results.

## 5 Conclusion

The findings of this study suggest that mutations in respiratory chain genes may elevate the risk of both the phylogenetic clustering and emergence of MDR-TB. This insight offers a novel perspective for preventing MDR-TB and underscores the potential of targeting the respiratory chain as a therapeutic strategy.

## Data Availability

The datasets presented in this study can be found in online repositories. The names of the repository/repositories and accession number(s) can be found in the article/**Supplementary Material**. Additionally, we have uploaded the code to the GitHub repository, which can be accessed at https://github.com/shenmemingziheshi/Statistical-code.git.
