# Supplementary material for: Respiratory chain gene mutations associated with global phylogenetic clustering of drug-resistant Mycobacterium tuberculosis revealed by whole-genome sequencing
Source: Front Immunol. 2026 May 20;17:1724194. doi: 10.3389/fimmu.2026.1724194 (PMC13229807; doi:10.3389/fimmu.2026.1724194)
Supplement: Supplementary file 5 [file Table5.docx]

Supplementary Material

# Supplementary Tables

**Supplementary Table 5.** The performance of various models for discriminating clustered isolates from non-clustered isolates in the MDR cohort.

| **Parameters** | **Training set** | | **Test set** | |
| --- | --- | --- | --- | --- |
|  | **(n=2835, 1391 clustered isolates, 1444 non-clustered isolates)** | | **(n=1216,596 clustered isolates,620 non-clustered isolates)** | |
|  | **Random Forest** | **Gradient Boosted Classification Tree** | **Random Forest** | **Gradient Boosted Classification Tree** |
| Kappa | 0.515 | 0.524 | 0.47 | 0.444 |
| AUC | 0.819 | 0.819 | 0.785 | 0.761 |
| (95% CI) | (0.805,0.833) | (0.805,0.833) | (0.762,0.808) | (0.737,0.785) |
| Sensitivity | 0.748 | 0.769 | 0.727 | 0.721 |
| (95% CI) | (0.732,0.764) | (0.753,0.785) | (0.702,0.752) | (0.696,0.746) |
| Specificity | 0.767 | 0.755 | 0.744 | 0.723 |
| (95% CI) | (0.751,0.783) | (0.739,0.771) | (0.719,0.769) | (0.698,0.748) |
| PPV | 0.755 | 0.75 | 0.731 | 0.719 |
| (95% CI) | (0.739,0.771) | (0.734,0.766) | (0.706,0.756) | (0.694,0.744) |
| NPV | 0.76 | 0.774 | 0.739 | 0.725 |
| (95% CI) | (0.744,0.776) | (0.759,0.789) | (0.714,0.764) | (0.7,0.75) |
| PLR | 3.145 | 3.316 | 2.8 | 2.618 |
| (95% CI) | (3.118,3.172) | (3.29,3.342) | (2.756,2.844) | (2.573,2.663) |
| NIR | 0.318 | 0.302 | 0.357 | 0.382 |
| (95% CI) | (0.251,0.385) | (0.236, 0.368) | (0.26,0.454) | (0.287,0.477) |
| Accuracy | 0.758 | 0.762 | 0.735 | 0.722 |
| (95% CI) | (0.742,0.774) | (0.746,0.778) | (0.71,0.76) | (0.697,0.747) |

AUC, area under the curve; PPV, positive predictive value; NPV, negative predictive value; PLR, positive likelihood ratio; NLR, negative likelihood ratio; CI, confidence.
