# Supplementary material for: Respiratory chain gene mutations associated with global phylogenetic clustering of drug-resistant Mycobacterium tuberculosis revealed by whole-genome sequencing
Source: Front Immunol. 2026 May 20;17:1724194. doi: 10.3389/fimmu.2026.1724194 (PMC13229807; doi:10.3389/fimmu.2026.1724194)
Supplement: Supplementary file 6 [file Table6.docx]

Supplementary Material

# Supplementary Tables

**Supplementary Table 6.** The performance of the random forest and gradient boosting models in discriminating between MDR and SDR isolates.

| **Parameters** | **Training set**  **(n=3566 2816 MDR isolates, 750 SDR isolates)** | | **Test set**  **(n=1529 1235 MDR isolates, 294 SDR isolates)** | |
| --- | --- | --- | --- | --- |
|  | **Random Forest** | **Gradient Boosted Classification Tree** | **Random Forest** | **Gradient Boosted Classification Tree** |
| Kappa | 0.493 | 0.506 | 0.422 | 0.525 |
| AUC | 0.845 | 0.843 | 0.805 | 0.828 |
| (95% CI) | (0.833,0.857) | (0.831,0.855) | (0.785,0.825) | (0.809,0.847) |
| Sensitivity | 0.984 | 0.976 | 0.985 | 0.964 |
| (95% CI) | (0.980,0.988) | (0.971,0.981) | (0.979,0.991) | (0.955,0.973) |
| Specificity | 0.415 | 0.441 | 0.34 | 0.489 |
| (95% CI) | (0.399,0.431) | (0.425,0.457) | (0.316,0.364) | (0.464,0.514) |
| PPV | 0.863 | 0.873 | 0.863 | 0.877 |
| (95% CI) | (0.852,0.874) | (0.862,0.884) | (0.846,0.880) | (0.861,0.893) |
| NPV | 0.871 | 0.827 | 0.847 | 0.78 |
| (95% CI) | (0.860,0.882) | (0.815,0.839) | (0.829,0.865) | (0.759,0.801) |
| PLR | 6.699 | 5.04 | 5.654 | 3.988 |
| (95% CI) | (6.682,6.716) | (5.018,5.062) | (5.624,5.684) | (3.944,4.032) |
| NIR | 0.149 | 0.198 | 0.177 | 0.251 |
| (95% CI) | (0.066,0.232) | (0.111,0.285) | (0.05,0.304) | (0.116,0.386) |
| Accuracy | 0.864 | 0.868 | 0.861 | 0.865 |
| (95% CI) | (0.853,0.875) | (0.857,0.879) | (0.844,0.878) | (0.848,0.882) |

AUC, area under the curve; PPV, positive predictive value; NPV, negative predictive value; PLR, positive likelihood ratio; NLR, negative likelihood ratio; CI, confidence.
