# Supplementary material for: Respiratory chain gene mutations associated with global phylogenetic clustering of drug-resistant Mycobacterium tuberculosis revealed by whole-genome sequencing
Source: Front Immunol. 2026 May 20;17:1724194. doi: 10.3389/fimmu.2026.1724194 (PMC13229807; doi:10.3389/fimmu.2026.1724194)
Supplement: Supplementary file 7 [file Table7.docx]

Supplementary Material

# Supplementary Tables

**Supplementary Table 7.** The performance of the random forest and gradient boosting models for discriminating MDR isolates from Sensitive isolates.

| **Parameters** | **Training set**  **(n=8187,2822 MDR isolates,5365 Sensitive isolates)** | | **Test set**  **(n=3509,1229 MDR isolates,2280 Sensitive isolates)** | |
| --- | --- | --- | --- | --- |
|  | **Random Forest** | **Gradient Boosted Classification Tree** | **Random Forest** | **Gradient Boosted Classification Tree** |
| Kappa | 0.65 | 0.618 | 0.606 | 0.598 |
| AUC | 0.936 | 0.926 | 0.922 | 0.92 |
| (95% CI) | (0.931,0.941) | (0.92,0.932) | (0.913,0.931) | (0.911,0.929) |
| Sensitivity | 0.68 | 0.655 | 0.644 | 0.641 |
| (95% CI) | (0.67,0.69) | (0.645,0.665) | (0.628,0.66) | (0.625,0.657) |
| Specificity | 0.939 | 0.932 | 0.93 | 0.926 |
| (95% CI) | (0.934,0.944) | (0.927,0.937) | (0.922,0.938) | (0.917,0.935) |
| PPV | 0.854 | 0.835 | 0.833 | 0.821 |
| (95% CI) | (0.846,0.862) | (0.827,0.843) | (0.821,0.845) | (0.808,0.834) |
| NPV | 0.848 | 0.836 | 0.829 | 0.83 |
| (95% CI) | (0.84,0.856) | (0.828, 0.844) | (0.817,0.841) | (0.818,0.842) |
| PLR | 5.612 | 5.094 | 4.865 | 4.82 |
| (95% CI) | (5.599,5.625) | (5.081,5.107) | (4.845,4.885) | (4.8,4.84) |
| NIR | 0.178 | 0.196 | 0.206 | 0.207 |
| (95% CI) | (0.125,0.231) | (0.146,0.246) | (0.131,0.281) | (0.135,0.279) |
| Accuracy | 0.85 | 0.836 | 0.83 | 0.827 |
| (95% CI) | (0.842,0.858) | (0.828,0.844) | (0.818,0.842) | (0.814,0.84) |

AUC, area under the curve; PPV, positive predictive value; NPV, negative predictive value; PLR, positive likelihood ratio; NLR, negative likelihood ratio; CI, confidence.
