# Supplementary material for: Respiratory chain gene mutations associated with global phylogenetic clustering of drug-resistant Mycobacterium tuberculosis revealed by whole-genome sequencing
Source: Front Immunol. 2026 May 20;17:1724194. doi: 10.3389/fimmu.2026.1724194 (PMC13229807; doi:10.3389/fimmu.2026.1724194)
Supplement: Supplementary file 8 [file Table8.docx]

Supplementary Material

# Supplementary Tables

# **Supplementary Table 8.** Analysis of generalized linear mixed model of MDR and sensitive isolates.

| **Gene** | **Position** | **SNP** | **Amino acid changes** | ***p-*Value** | **OR(95%CI)** |
| --- | --- | --- | --- | --- | --- |
| hycE | 95591 | G178T | Glu60* | 0.207 | 0.518(0.187-1.438) |
| hycE | 95635 | G222A | Ala74Ala | 0.062 | 0.053(0.002-1.157) |
| hycE | 95662 | C249T | Phe83Phe | 0.706 | 1.59(0.144-17.613) |
| hycE | 95697 | C284T | Pro95Leu | 0.942 | NA |
| hycE | 95812 | G399A | Pro133Pro | 0.777 | 0.861(0.305-2.432) |
| hycE | 96007 | C594T | Leu198Leu | 0.986 | NA |
| ndhA | 471469 | G1171A | Gly391Arg | 0.999 | NA |
| ndhA | 471612 | A1028G | Tyr343Cys | <0.001 | 0.223(0.107-0.464) |
| ndhA | 471616 | C1024T | Arg342* | 0.128 | 0.081(0.003-2.055) |
| ndhA | 471640 | G1000A | Ala334Thr | <0.001 | 17.047(4.356-66.723) |
| ndhA | 471646 | G994C | Gly332Arg | 0.800 | 0.875(0.311-2.462) |
| ndhA | 471666 | T974C | Met325Thr | 0.998 | NA |
| ndhA | 472148 | T492C | His164His | 0.941 | 1.106(0.076-15.992) |
| ndhA | 472236 | C404A | Ala135Glu | 0.908 | 0.95(0.398-2.27) |
| ndhA | 472471 | C169T | Gln57* | 0.999 | NA |
| ndhA | 472534 | G106C | Ala36Pro | 0.999 | NA |
| atpB | 1460552 | G309T | Trp103Cys | 0.975 | NA |
| atpB | 1460907 | T664C | Phe222Leu | 0.895 | 1.094(0.286-4.184) |
| atpB | 1460992 | A749C | His250Pro | 0.856 | 1.18(0.196-7.1) |
| atpF | 1461414 | T94C | Phe32Leu | 0.949 | NA |
| atpH | 1461915 | C73G | Leu25Val | 0.999 | NA |
| atpH | 1462152 | C310A | Arg104Ser | 0.102 | 1.418(0.933-2.156) |
| atpH | 1462181 | G339A | Leu113Leu | 1.000 | NA |
| atpH | 1462270 | A428G | Gln143Arg | 1.000 | NA |
| atpH | 1462713 | G871A | Glu291Lys | 1.000 | NA |
| atpH | 1462787 | C945A | Asp315Glu | 0.323 | 1.43(0.703-2.91) |
| atpH | 1462845 | G1003A | Asp335Asn | 0.145 | 2.927(0.691-12.403) |
| atpH | 1463106 | A1264G | Ile422Val | 0.984 | NA |
| atpA | 1463498 | G271C | Val91Leu | 0.016 | 6.437(1.406-29.475) |
| atpA | 1463923 | C696T | Thr232Thr | 0.031 | 0.166(0.032-0.853) |
| atpA | 1464070 | C843T | Tyr281Tyr | 0.268 | 2.17(0.551-8.555) |
| atpA | 1464223 | C996T | Thr332Thr | 0.487 | 0.553(0.104-2.932) |
| atpA | 1464859 | G1632A | Ala544Ala | 0.943 | NA |
| cydB | 1823378 | G1023A | Leu341Leu | <0.001 | 0.084(0.022-0.329) |
| cydB | 1823579 | G822A | Leu274Leu | 0.120 | 0.212(0.03-1.5) |
| cydB | 1823738 | G663A | Leu221Leu | 0.074 | 4.129(0.87-19.602) |
| cydB | 1823798 | C603T | Ala201Ala | 1.000 | NA |
| cydB | 1823818 | G583C | Asp195His | 1.000 | NA |
| cydB | 1823906 | C495T | Tyr165Tyr | 0.999 | NA |
| cydB | 1824097 | G304A | Ala102Thr | 0.601 | 0.458(0.025-8.54) |
| cydB | 1824275 | T126C | Asp42Asp | 0.046 | 4.415(1.027-18.988) |
| cydA | 1824493 | G1395A | Ala465Ala | 1.000 | NA |
| cydA | 1824726 | A1162G | Asn388Asp | 0.343 | 0.307(0.027-3.533) |
| cydA | 1824800 | G1088A | Arg363His | 0.011 | 46.712(2.398-909.959) |
| cydA | 1824946 | C942A | Ile314Ile | <0.001 | 15.627(5.405-45.184) |
| cydA | 1825046 | G842A | Arg281Gln | 0.993 | NA |
| cydA | 1825857 | T31C | Phe11Leu | 0.999 | NA |
| ctaE | 2456913 | G13A | Val5Ile | 1.000 | NA |
| qcrC | 2457664 | G112C | Ala38Pro | 0.974 | NA |
| qcrC | 2457819 | G267A | Pro89Pro | 1.000 | NA |
| qcrC | 2458003 | G451C | Gly151Arg | 1.000 | NA |
| qcrC | 2458234 | A682C | Lys228Gln | 1.000 | NA |
| qcrC | 2458368 | G816C | Leu272Leu | 0.280 | 5.191(0.262-102.728) |
| qcrA | 2458572 | G181C | Glu61Gln | 0.991 | NA |
| qcrA | 2458639 | T248C | Val83Ala | 0.083 | 2.199(0.903-5.354) |
| qcrA | 2458723 | T332C | Leu111Ser | 1.000 | NA |
| qcrA | 2459162 | G771A | Glu257Glu | 0.108 | 0.103(0.007-1.641) |
| qcrB | 2459732 | G55A | Glu19Lys | 0.362 | 1.524(0.615-3.776) |
| qcrB | 2460254 | T577G | Trp193Gly | 0.372 | 0.68(0.292-1.584) |
| qcrB | 2460407 | C730A | Pro244Thr | 0.997 | NA |
| qcrB | 2460628 | C951A | Ala317Ala | 0.999 | NA |
| qcrB | 2460755 | A1078G | Thr360Ala | 0.995 | NA |
| qcrB | 2460927 | G1250T | Arg417Leu | 1.000 | NA |
| qcrB | 2461053 | C1376A | Pro459His | 0.997 | NA |
| ctaC | 2464442 | A310C | Thr104Pro | 0.914 | 0.932(0.26-3.338) |
| ctaC | 2464458 | G294C | Arg98Arg | 0.999 | NA |
| ctaD | 3403221 | C1701T | Pro567Pro | 1.000 | NA |
| ctaD | 3403250 | G1672A | Ala558Thr | 0.675 | 0.703(0.135-3.655) |
| ctaD | 3403254 | C1668T | Ala556Ala | 0.360 | 0.158(0.003-8.202) |
| ctaD | 3403436 | T1486C | Phe496Leu | 0.570 | 0.655(0.152-2.818) |
| ctaD | 3403496 | G1426T | Val476Phe | 0.341 | 0.359(0.044-2.959) |
| ctaD | 3404178 | C744A | Ala248Ala | 0.997 | NA |
| ctaD | 3404254 | C668T | Ala223Val | 1.000 | NA |
| ctaD | 3404331 | C591A | Arg197Arg | 0.958 | NA |
| ctaD | 3404376 | G546C | Thr182Thr | <0.001 | 0.581(0.429-0.789) |
| ctaD | 3404689 | A233G | Gln78Arg | 0.443 | 0.528(0.103-2.704) |
| ctaD | 3404883 | C39T | Ala13Ala | 0.999 | NA |
| nuoA | 3511716 | C35T | Ala12Val | 0.384 | 4.263(0.162-111.868) |
| nuoA | 3511763 | G82C | Val28Leu | <0.001 | 3.364(1.84-6.15) |
| nuoA | 3511965 | A284G | Asp95Gly | 0.158 | 3.935(0.589-26.297) |
| nuoB | 3512352 | G276A | Ala92Ala | 0.999 | NA |
| nuoB | 3512566 | G490C | Ala164Pro | <0.001 | 6.835(2.905-16.083) |
| nuoC | 3512634 | C7T | Pro3Ser | 0.590 | 0.483(0.034-6.823) |
| nuoD | 3514512 | G1175C | Gly392Ala | <0.001 | 0.358(0.22-0.582) |
| nuoE | 3514777 | G121C | Glu41Gln | 0.630 | 1.458(0.315-6.755) |
| nuoE | 3515307 | G651A | Gln217Gln | 0.960 | NA |
| nuoF | 3515467 | C56T | Pro19Leu | 0.998 | NA |
| nuoF | 3515477 | G66C | Trp22Cys | 0.969 | NA |
| nuoF | 3515582 | C171T | Ser57Ser | 0.004 | 5.197(1.678-16.092) |
| nuoF | 3515936 | C525T | His175His | 0.046 | 0.168(0.029-0.966) |
| nuoF | 3516039 | G628A | Ala210Thr | 0.997 | NA |
| nuoF | 3516105 | A694G | Ile232Val | 0.954 | NA |
| nuoF | 3516161 | C750T | Phe250Phe | 0.916 | NA |
| nuoF | 3516342 | C931T | Leu311Leu | 0.964 | 1.02(0.441-2.356) |
| nuoG | 3517413 | C668T | Ala223Val | 0.077 | 0.221(0.042-1.18) |
| nuoG | 3518089 | T1344C | Gly448Gly | 0.003 | 0.015(0.001-0.241) |
| nuoG | 3518167 | A1422G | Ile474Met | <0.001 | 3.401(2.344-4.934) |
| nuoG | 3518371 | T1626C | His542His | 0.972 | NA |
| nuoG | 3518392 | G1647A | Ala549Ala | 0.853 | 0.803(0.078-8.254) |
| nuoG | 3518441 | T1696C | Leu566Leu | 0.105 | 1.862(0.879-3.943) |
| nuoG | 3518555 | A1810G | Thr604Ala | <0.001 | 0.101(0.052-0.197) |
| nuoG | 3518993 | G2248A | Asp750Asn | 1.000 | NA |
| nuoG | 3519161 | T2416C | Ser806Pro | 0.999 | NA |
| nuoH | 3519636 | C355T | Leu119Phe | 0.331 | 1.848(0.536-6.37) |
| nuoH | 3519732 | C451T | Leu151Leu | 1.000 | NA |
| nuoH | 3519774 | G493A | Val165Ile | 0.001 | 0.49(0.321-0.748) |
| nuoH | 3520257 | G976A | Val326Ile | 1.000 | NA |
| nuoH | 3520485 | G1204T | Gly402Cys | 0.999 | NA |
| nuoI | 3520977 | G471C | Leu157Leu | 0.688 | 0.926(0.636-1.348) |
| nuoI | 3521044 | A538G | Thr180Ala | 0.999 | NA |
| nuoJ | 3521253 | G115A | Val39Ile | 1.000 | NA |
| nuoJ | 3521432 | G294A | Leu98Leu | <0.001 | 0.008(0.002-0.036) |
| nuoJ | 3521639 | T501C | Ile167Ile | 0.966 | NA |
| nuoK | 3521996 | C73T | Arg25Cys | <0.001 | 28.876(5.822-143.211) |
| nuoK | 3522012 | T89C | Met30Thr | 0.063 | 0.23(0.049-1.082) |
| nuoL | 3522395 | G162A | Leu54Leu | 0.998 | NA |
| nuoL | 3523741 | C1508A | Thr503Asn | 0.989 | NA |
| nuoM | 3524186 | G55C | Val19Leu | 1.000 | NA |
| nuoM | 3524488 | G357C | Arg119Ser | 0.588 | 0.515(0.047-5.663) |
| nuoM | 3524528 | G397T | Gly133Cys | <0.001 | 0.222(0.135-0.365) |
| nuoM | 3524581 | C450T | Ile150Ile | 0.158 | 4.74(0.548-41.016) |
| nuoM | 3524903 | G772A | Ala258Thr | <0.001 | 0.154(0.086-0.276) |
| nuoM | 3524923 | G792A | Pro264Pro | 0.401 | 7.257(0.071-745.071) |
| nuoM | 3525374 | A1243G | Thr415Ala | 0.434 | 1.911(0.377-9.684) |
| nuoN | 3526137 | T348G | Ala116Ala | 0.313 | 0.606(0.229-1.605) |
| nuoN | 3526539 | G750A | Ala250Ala | 0.998 | NA |
| nuoN | 3526572 | A783G | Leu261Leu | 0.998 | NA |
| nuoN | 3526873 | G1084T | Ala362Ser | <0.001 | 2.726(1.879-3.955) |
| nuoN | 3526884 | G1095A | Pro365Pro | 0.372 | 2.386(0.354-16.094) |
| nuoN | 3526986 | A1197G | Ala399Ala | 0.999 | NA |
| nuoN | 3526987 | G1198A | Gly400Ser | 0.911 | 1.205(0.045-31.982) |

SNP, single nucleotide polymorphism; OR, odd ratio; CI, confident interval; NA, not available.
