# Supplementary material for: Respiratory chain gene mutations associated with global phylogenetic clustering of drug-resistant Mycobacterium tuberculosis revealed by whole-genome sequencing
Source: Front Immunol. 2026 May 20;17:1724194. doi: 10.3389/fimmu.2026.1724194 (PMC13229807; doi:10.3389/fimmu.2026.1724194)
Supplement: Supplementary file 12 [file Table12.docx]

Supplementary Material

# Supplementary Figures

For more information on Supplementary Material and for details on the different file types accepted, please see [here](https://www.frontiersin.org/guidelines/author-guidelines#supplementary-material).

**
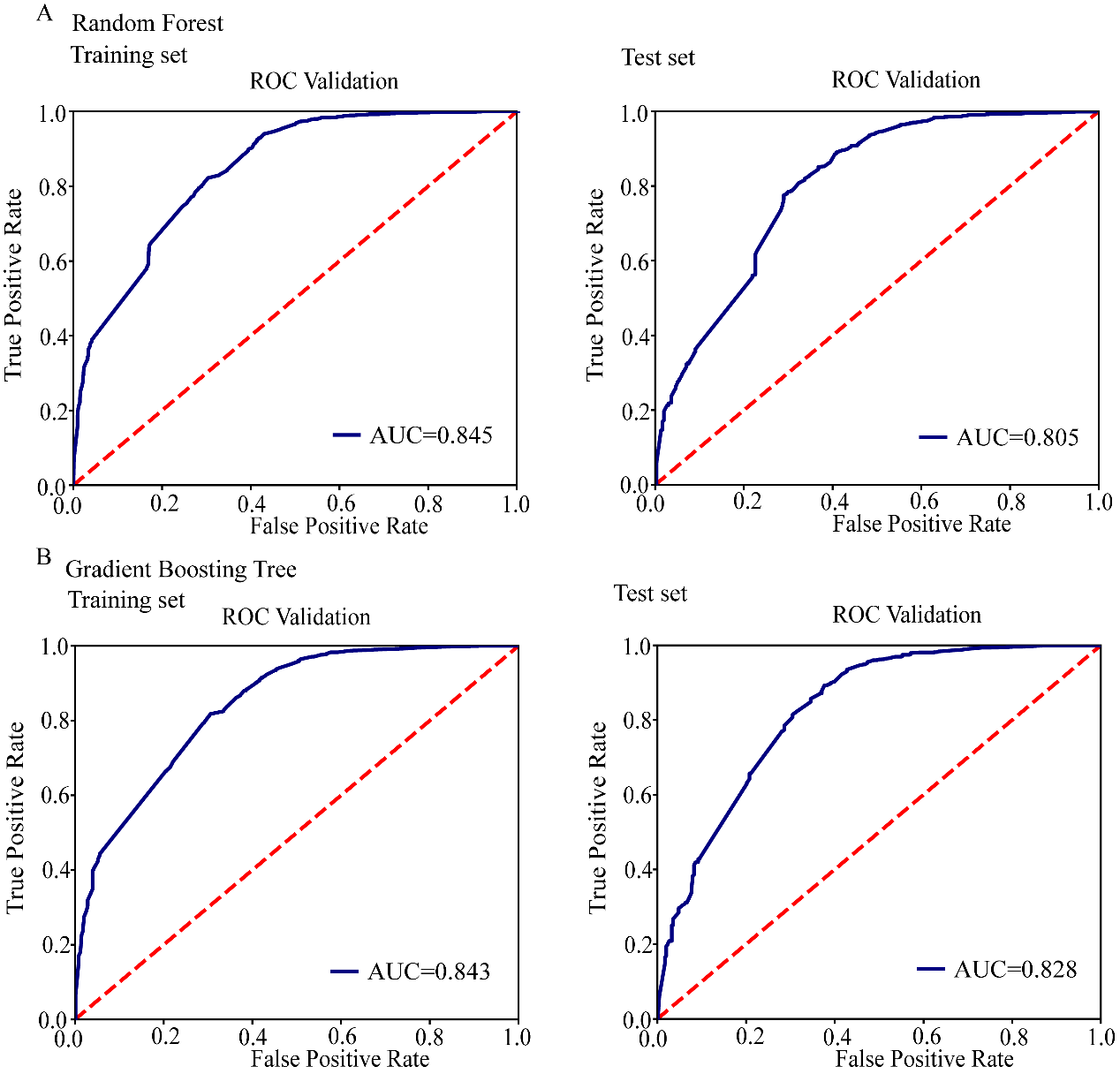
**

**Supplementary Figure 1.** ROC curve analysis was conducted to evaluate the performance of models used for discriminating MDR isolates from SDR isolates. (A) ROC analysis showing the performance of the random forest model. (B) ROC analysis showing the performance of the gradient boosting decision tree.


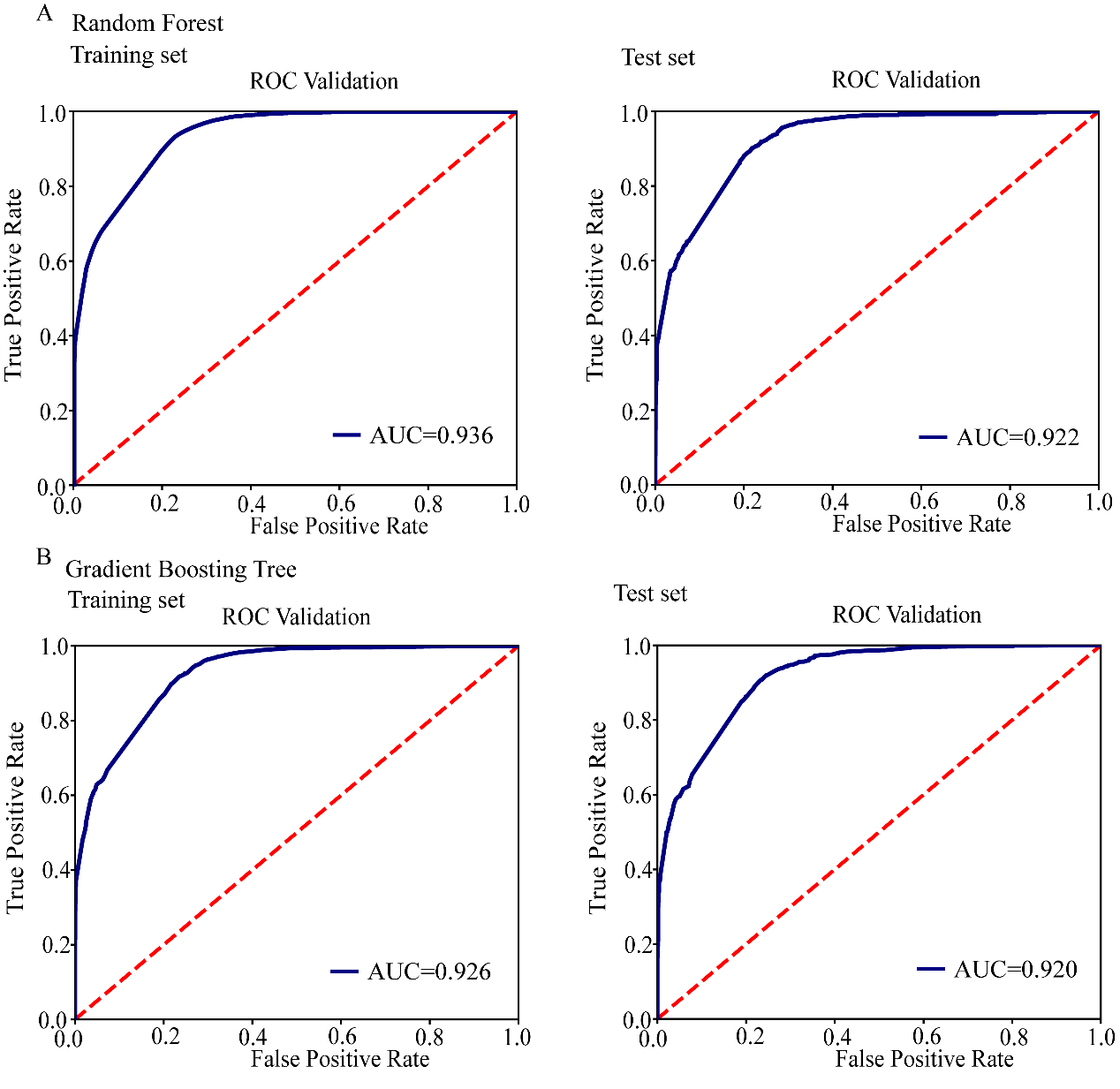


**Supplementary Figure 2.** ROC curve analysis was conducted to evaluate the performance of models used for discriminating MDR isolates from sensitive isolates. (A) ROC analysis showing the performance of the random forest model. (B) ROC analysis showing the performance of the gradient boosting decision tree.


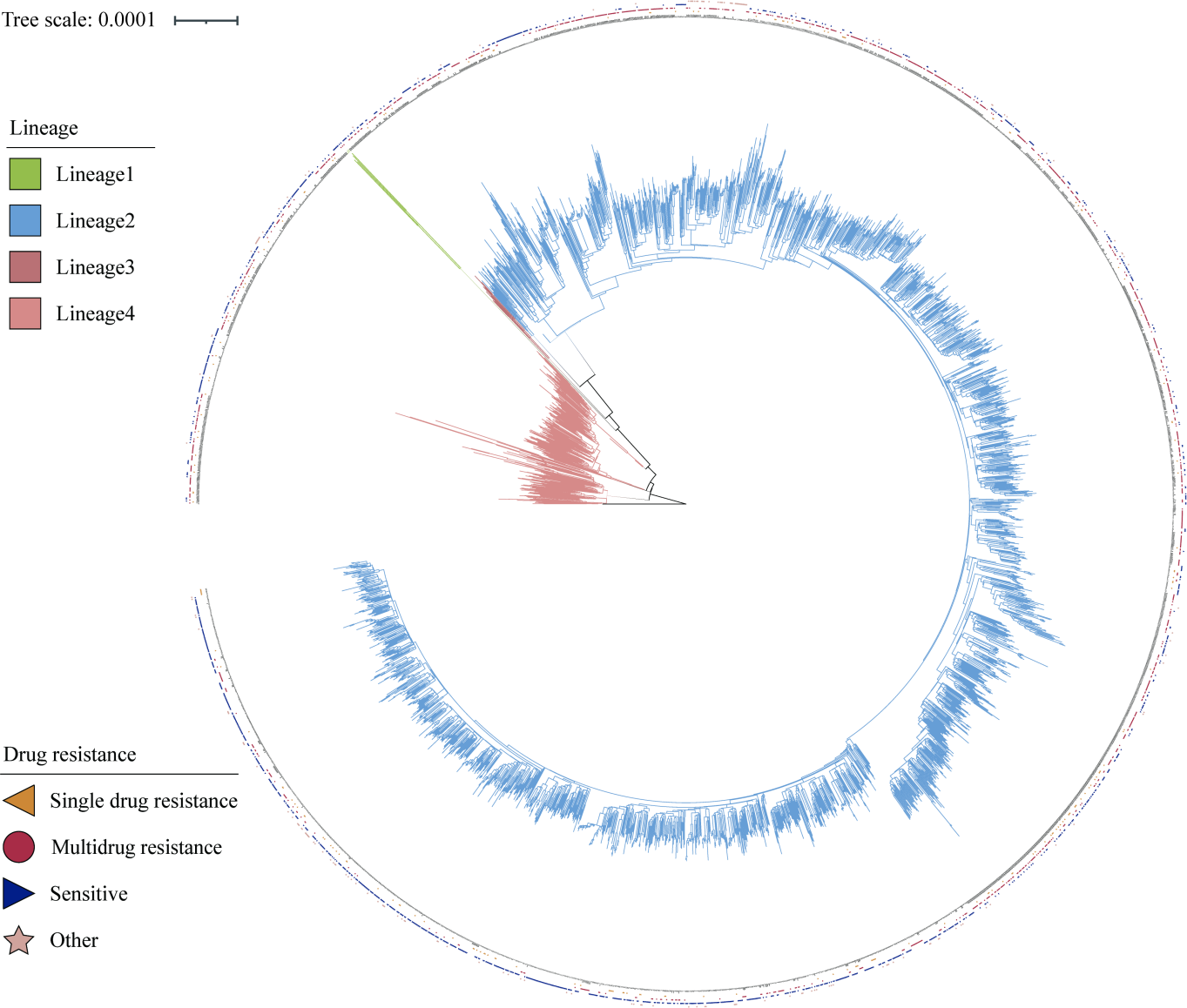


**Supplementary Figure 3.** The phylogenetic tree of *Mycobacterium tuberculosis* isolates from China.
